# 6-shogaol is a potential treatment for Head and Neck Squamous Cell Carcinoma

**DOI:** 10.7150/ijms.80542

**Published:** 2023-01-22

**Authors:** Cheng-Ming Hsu, Hui-Chen Su, Ming-Yu Yang, Yao-Te Tsai, Ming-Shao Tsai, Yao-Hsu Yang, Ching-Yuan Wu, Shun-Fu Chang

**Affiliations:** 1 Department of Otolaryngology-Head and Neck Surgery, Chiayi Chang Gung Memorial Hospital, Chiayi, Taiwan.; 2 School of Medicine, College of Medicine, Chang Gung University, Taoyuan, Taiwan.; 3 Cancer Center, Chiayi Chang Gung Memorial Hospital, Chiayi, Taiwan.; 4 Department of Neurology, National Cheng-Kung University Hospital, College of Medicine, National Cheng Kung University, Tainan, Taiwan.; 5 Graduate Institute of Clinical Medical Sciences, College of Medicine, Chang Gung University, Taoyuan, Taiwan.; 6 Department of Otolaryngology, Kaohsiung Chang Gung Memorial Hospital, Kaohsiung, Taiwan.; 7 Department of Chinese Medicine, Chiayi Chang Gung Memorial Hospital, Chiayi, Taiwan.; 8 School of Chinese Medicine, College of Medicine, Chang Gung University, Taoyuan 33302, Taiwan.; 9 Department of Medical Research and Development, Chiayi Chang Gung Memorial Hospital, Chiayi, No 6, Sec. West, Jiapu Rd., Puzi-City, Chiayi County, 61363, Taiwan.

**Keywords:** 6-shogaol, head and neck squamous cell carcinoma, apoptosis, ginger, p38 signaling, extracellular signal-regulated kinase, mammalian mitogen-activated protein kinases

## Abstract

**Objective:** Natural products in diet have shown a potential role in the prevention and treatment of cancer. Ginger (Zingiber officinale Roscoe) is a great candidate because of its properties of anti-inflammatory, antioxidant, and anti-cancer, but little is known about its effect on head and neck cancer. 6-Shogaol is an active compound derived from Ginger. Thus, this study aimed to investigate the possible anticancer effects of 6-shogaol, a major ginger derivate, on head and neck squamous cell carcinomas (HNSCCs) and the underlying mechanisms.

**Material and Methods:** Two HNSCC cell lines, SCC4 and SCC25, were used in this study. Both SCC4 and SCC25 cells were kept as control or treated with 6-shogaol for 8 and 24 hours and then the cell apoptosis and cell cycle progression of treated cells were examined by PI and Annexin V-FITC double stain and flow cytometry analysis. The Cleaved caspase 3, phosphorylations of ERK1/2 and p38 kinases were examined by Western blot analysis.

**Results:** The results showed that 6-shogaol significantly initiated the G2/M phase arrest of the cell cycle and apoptosis to inhibit the survival of both cell lines. Moreover, these responses could be regulated by ERK1/2 and p38 signaling. And, finally, we also demonstrated that 6-shogaol could enhance the cytotoxicity of cisplatin in HNSCC cells.

**Conclusion:** Our data provided new insights to understand the potential pharmaceutical efficacy of a ginger derivate, 6-shogaol, in antagonizing HNSCC survival. The present study suggests that 6-shogaol is a potential novel candidate for anti-HNSCCs therapy.

## Introduction

Head and neck squamous cell carcinomas (HNSCCs), the most aggressive and malignant cancer types in head and neck tissues, are epithelial cancers and majorly occur in the oral cavity, larynx, and pharynx regions. According to recent statistical data, more than 800 thousand patients with HNSCCs have been found in clinical diagnoses every year around the world 1,2. In Taiwan, HNSCC is also the sixth most common cancer and the 5th leading cause of death. Moreover, it has been shown that approximately 7,300 patients are diagnosed with HNSCCs per year, and 2,200 patients die of the disease in Taiwan 3. The current clinical standard treatments for HNSCC patients include surgery, radiation, chemotherapy, and even a combination of these options 4. However, these non-selective therapies can lead to relevant complications, and often produce much systemic toxicity. Moreover, more than 40% of patients might suffer from cancer recurrence 5,6. All above are the possible reasons that contribute to the sustainedly low overall survival rate 1,2. The pathogenic factors of HNSCCs varied across the regions, however, the major factors include tobacco and alcohol consumption and the infection of human papillomavirus (HPV). Moreover, it has been further found that most of the HNSCC patients are labor class with a lower socioeconomic status. Therefore, a more understanding of HNSCC development and the underlying mechanism is still crucial and this might provide new knowledge and ideasto improve the clinical outcome of HNSCCs.

Accumulating evidence has proved that many natural products in the diet is a great source of medication to prevent and treat cancer effectively 7,8. Furthermore, it has also been shown that the large number of components from dietetic therapy could potentially reduce relapses and improve cancer survival 7. Various epidemiological studies have eludicated that the consumption of soy products, fruits, spices, and vegetables (especially cruciferous vegetables) is associated with a reduced risk of cancer occurrence. Ginger is a rhizome of a sterile plant, which attracts much attention because of its antioxidant, anti-inflammatory, and anti-cancer properties. Ginger has been widely used in diet and traditional herbal spices around the world 9,10. In the aspect of medicine, it has been applied in the treatment of indigestion, nausea, vomiting, pain, common cold, infection or inflammation, and diarrhea. Moreover, it was proved that ginger had effectiveness on colon cancer 11, cervical cancer 12, non-small cell cancer 13, breast cancer 14, and prostate adenocarcinoma 15,16. From the ingredients analysis, phenolic and terpene compounds were thought to be the major functional constituents of ginger 17. Gingerol and shogaol are two polyphenols; more and more studies had found that both gingerol and shogaol derivates, e.g., 6-shogaol, could improve the invasion and metastasis of cancer through different molecular mechanisms. 6-Shogaol, the dehydrated 6-gingerol, extracted from Ginger, possessed much stronger anti-tumor activity than 6-gingerol 18,19.

Recent literature further showed that 6-shogaol could trigger the apoptosis of HNSCCs and increase radiation sensitivity of cancer cells as well 20. However, there are fewer studies on its underlying mechanism. The aim of this study was to further investigate the effect and the underlying mechanism of 6-shogaol on the HNSCCs. It had been believed that 6-Shogaol could arrest the cell cycle in two types of HNSCC cells, i.e., SCC4 and SCC25, at the G2/M phase and promote apoptosis to inhibit their survival through ERK1/2 and p38 signaling pathways. Cisplatin-based concurrent chemoradiation is the standard of treatment for patients with locoregionally advanced HNSCC 4. Moreover, our result also found that the combination of 6-shogaol with cisplatin could enhance the cisplatin cytotoxicity of HNSCC cells.

The present study not only figures out the anticancer mechanism of 6-shogaol, but also illustrates the potential role of 6-shogaol in the field of combination therapy for HNSCC patients, e.g., cisplatin, and 6-shogaol-including dietetic therapy.

## Materials and methods

### Cell culture and treatment

Two human HNSCC cell lines (tongue squamous cell carcinoma cell lines), SCC-4 and SCC25, used in this study were purchased from Food Industry Research and Development Institute, Taiwan. These cells were maintained in MEM (Minimum Essential Medium Eagle)-F12 medium (Invitrogen, Carlsbad, CA, USA) containing 0.4 μg/mL hydrocortisone (Sigma-Aldrich, St. Louis, MO, USA), 1X Antibiotic-antimycotic 1X solution (Gibco/Thermo Fisher Scientific, Waltham, MA, USA) and 10% FBS and were grown at 37 °C with 5% CO2. 6-shogaol was purchased from Toronto Research Chemicals, Toronto, ON, Canada.

### MTT assay

In brief, after treatment of 6-shogaol (3, 5, 10, 15, and 30 µM) for 24 and 48 hours, the cell culture media were replaced with a fresh medium containing 0.02% MTT (Sigma-Aldrich) and incubated for 2 hours; subsequently, the medium was replaced with 200 µL dimethyl sulfoxide with the density of 6,000 cells/well. The percentages of metabolically active cells were determined based on the mitochondrial conversion of MTT (3-[4,5-dimethylthiazol-2-yl]-2,5 diphenyl tetrazolium bromide) into formazine. The results were assessed in a 96-well format plate reader by measuring the absorbance at a wavelength of 570 nm on a DTX880 Multimode Detector (Beckman Coulter, Brea, CA, USA). All experiments were performed in triplicate.

### Wound-Healing Assay

The wound-healing assay was used to analyze the migration activity of cells. Before the application of the wound-healing assay, SCC-4 and SCC-25 cells were well cultured to ensure a homogeneous and viable cell monolayer. One day before the assay, 2 × 10^5^ cells were seeded in, and when cell confluence reached approximately 90%, a straight-edged, cell-free zone across the cell monolayer in each well. It was artificially created on the monolayer by a sterile, plastic, 200-µL-micropipette tip. After the straight wound was created, cells were washed with PBS to remove debris. Cells that had migrated into the wounded area for 16 hours were photographed using a Zeiss microscope (Zeiss, Gottingen, Germany) at 40× magnification, and the migration area was calculated using ImageJ free software, version 1.41o (NIH, Bethesda, MD, USA).

### PI stain (cell cycle) and Annexin V-FITC/PI double stain (necrosis and apoptosis) for Flow Cytometry

Two human HNSCC cell lines were seeded in a 100-mm plate and cultured overnight before treatment. After 6-shogaol (15 and 30 mM) treatment, the cell cycle progression and necrosis/apoptosis of the cells were detected by PI stain and annexin V/PI double stain according to the manufacturer's instructions. The stained samples were further analyzed using flow cytometry (BD Bioscience FacsCanto II Flow Cytometer, Marshall Scientific, Hampton, NH, USA).

### Western Blot Analysis

Lysis buffer (20 mM Tris-HCl at pH 7.5, 150 mM NaCl, 1 mM Na2EDTA, 1% Nonidet P-40 (NP-40), 1 mM ethylene glycol tetra-acetic acid (EGTA), 1% sodium deoxycholate,1 mM β-glycerophosphate, 1mM Na3VO4, 2.5 mM sodium pyrophosphate, and 1 µg/mL leupeptin) was added to samples for protein extraction. For Western blot, 30 µg of the total lysates were separated using 6% to 15% sodium dodecyl sulfate-polyacrylamide gel electrophoresis and transferred to a polyvinylidene fluoride membrane (Millipore, Darmstadt, Germany). After blocking with dried nonfat milk for 1 h, the membrane w-as incubated overnight with primary antibodies at 1:3000 dilution. The primary antibodies and antibodies against phosphorylated epitopes used in this study were β-actin, cleaved caspase 3, p-p38 kinase, and p-ERK1/2 kinase (Thr202/Tyr204) (all purchased from Cell Signaling Technologies, Danvers, MA, USA). β-actin (1:5000 dilution; Sigma Aldrich, St. Louis, MO, USA) was used as the internal control. Horseradish-peroxidase (HRP)-conjugated goat anti-mouse IgG (Sigma Aldrich, St. Louis, MO, USA) and goat anti-rabbit IgG (Sigma Aldrich, St.Louis, MO, USA) were used as secondary antibodies. Western Lightning® Plus-Enhanced Chemiluminescence (ECL) Substrates (PerkinElmer, Inc., Boston, MA, USA) were used to visualize the proteins.

### Statistical analyses for cell line studies

All values were the means ± standard error of the mean (SEM) of the replicate samples (n = 3 to 6, depending on the experiment), and experiments have repeated a minimum of three times. Differences between two groups were assessed using the unpaired two-tailed Student's t-test or by ANOVA if more than two groups were analyzed. The Tukey test was used as a post-hoc test in ANOVA for testing the significance of pairwise group comparisons. P-values < 0.05 were considered statistically significant in all comparisons. SPSS version 15.0 (SPSS, Chicago, IL, USA) was used for all calculations.

## Results

### 6-shogaol or combination with Cisplatin induces cell death of SCC4 and SCC25

**Figure 1:**
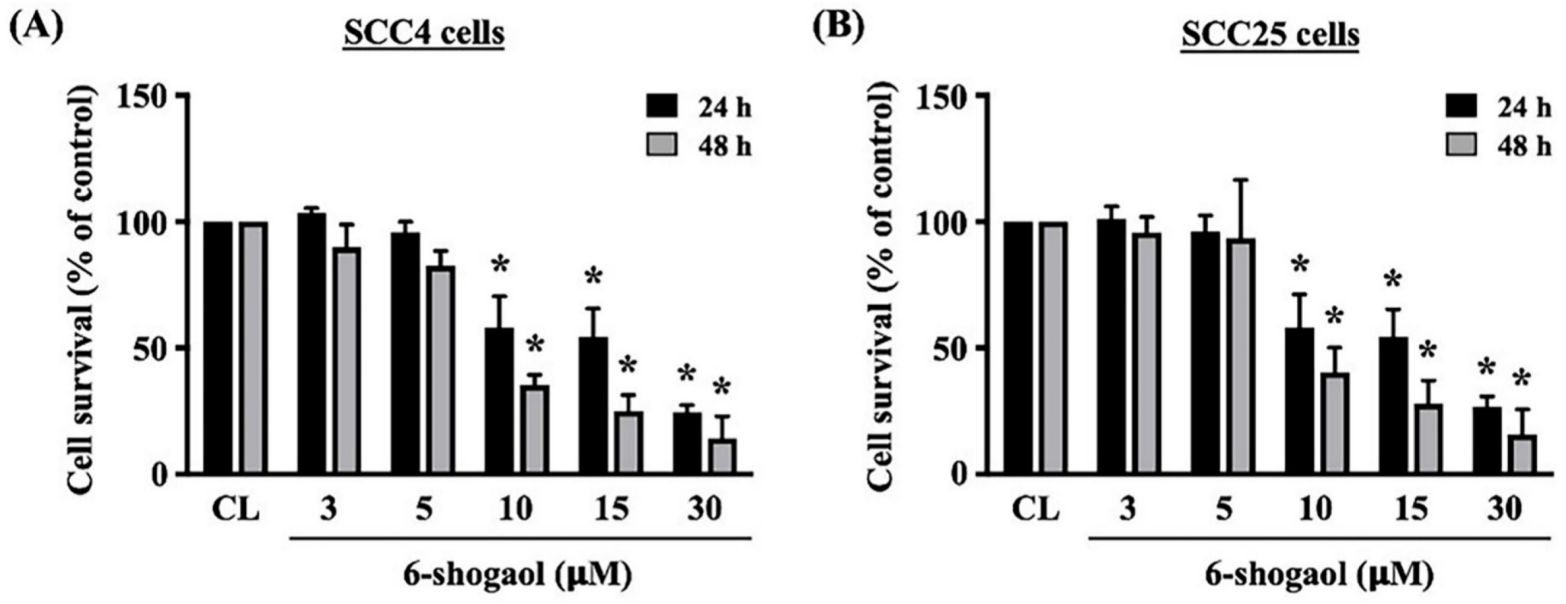
**6-shogaol induces cell death of A. SCC-4 and B. SCC-25 cells in the dose- and time-dependent manners.** The presented data are the means and standard errors of the mean of three independent experiments. * Indicates a statistically significant difference compared with untreated control cells of the same treatment duration. Two replicates of n = 4 independent experiments (# p < 0.001, * p < 0.05 by unpaired two tailed t-test).

Both HNSCC cell lines, i.e., SCC4 and SCC25, were kept as control or treated with 6-shogaol (3, 5, 10, 15, 30 mM) for 24 and 48 hours and then the viability of treated cells was checked by MTT assay. It revealed that 6-shogaol significantly results in the cell death of both cancer cells in dose-dependent and time-dependent manners compared to untreated control cells (Fig. 1A). The antiproliferative potential of 6-shogaol was assessed using MTT assay on SCC-4, and SCC-25. After 24 hours and 48 hours of treatment, the 10 μM 6-shogaol effectively inhibited the proliferation of both tongue cancer cells (SCC-4 and SCC-25) (Fig. 1).

### Low dose 6-shogaol can inhibit migration of HNSCC cell

**Figure 2:**
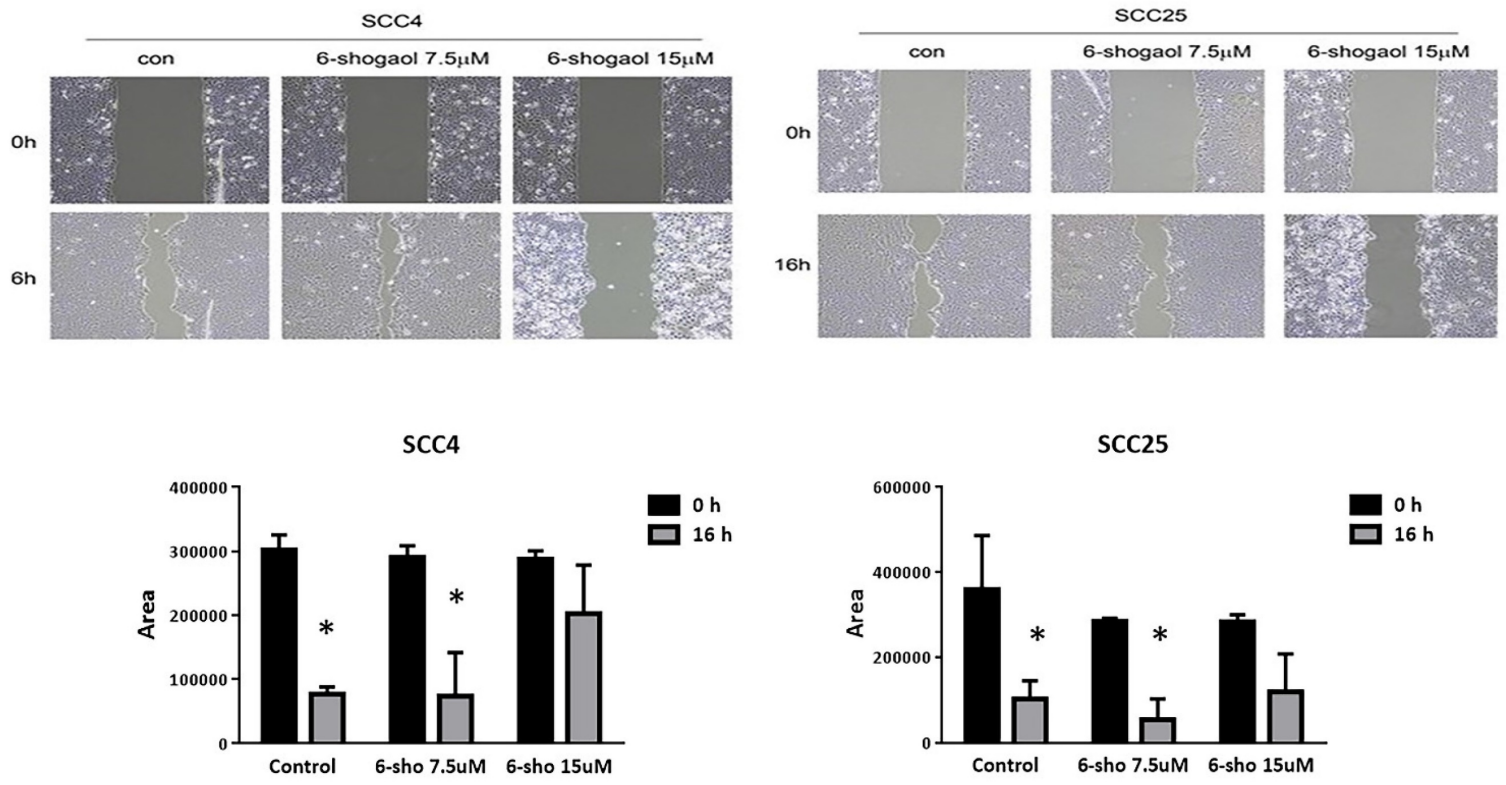
** The wound healing assay at 7.5 and 15μM. 6-shogaol (7.5 and 15 μM) was able to reduce wound closure.** Data were shown as mean ± SEM from three independent experiments. *P < 0.05 versus untreated control.

We performed a wound healing assay. As shown in Figure 2, 6-shogaol (7.5 and 15 μM) was able to reduce wound closure, by evaluating the percentage of the wound (compared to the respective T0) of 60% for SCC-4, against the 10% of the control, and 70% for SCC-25 compared to 25% of the control. 6-shogaol inhibited cell migration of SCC4 and SCC25 cells at 7.5 μM and induced cell death at 15 μM.

### 6-shogaol can induce cell cycle arrest in the G2/M phase

Cell cycle arrest at the G2/M phase in cancer cells is one of the anticancer mechanisms of many clinical drugs. Hence, we wondered if the cancer-killing effect of 6-shogaol on HNSCCs also stops the cell cycle progression. Both SCC4 and SCC25 cells were kept as control or treated with 6-shogaol (15 and 30 mM) for 8 and 24 hours and then the cell cycle progression of treated cells was examined by PI stain and flow cytometry analysis. Cells treated with 6-shogaol could result in changes in cell cycle progression at the G0/G1 and G2/M phases. Obviously, 15 mM 6-shogaol significantly induces cell cycle arrest at the G2/M phase in both SCC4 and SCC25 cells in time-dependent (Table 1) and dose-dependent (15 and 30 mM) (Table 2) manners compared to the untreated controls. These results suggest that 6-shogaol may enhance the cytostatic effect by promoting G2/M phase accumulation and inhibiting cell cycle progression.

**Figure 3:**
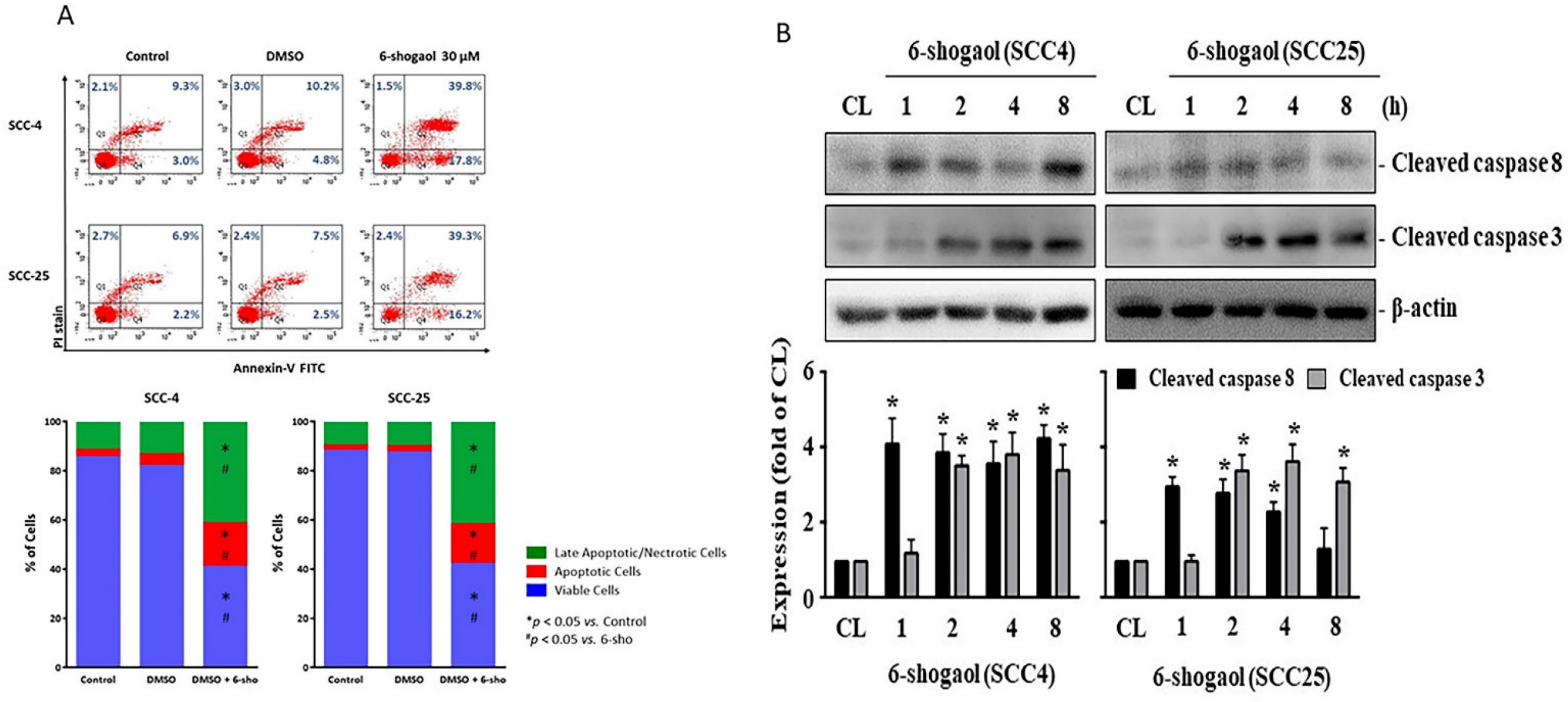
** 6-shogaol induces apoptosis of SCC4 and SCC25 cells. (A)** SCC4 and SCC25 cells were kept as control or treated with 6-shogaol (15 and 30 μM) for 24 h and then the cell apoptosis was examined by PI/Annexin V-FITC double stain and flow cytometry analysis. (Q1+Q2) was indicated as the late apoptotic and necrotic cells, Q3 was indicated as the living cells, and Q4 was indicated as the apoptotic cells. **(B)** SCC4 and SCC25 cells were kept as control or treated with 6-shogaol (10 μM) for 1, 2, 4, and 8 h and then the level of cleaved caspase 3 was examined by Western blot. Data in (A) were shown as mean ± SEM from three independent experiments. Results in (B) were representative of three independent experiments with similar results. *P < 0.05 versus untreated control.

On the other hand, we examined that if 6-shogaol elicits apoptosis of both SCC4 and SCC25 resulting in cell death. Both cells were kept as control or treated with 6-shogaol (15 and 30 mM) for 24 hours and then the cell apoptosis was examined by PI and Annexin V-FITC double stain and flow cytometry analysis. Cell apoptosis significantly developed in both cells treated with 6-shogaol, which initiated a decrease in living cells and an increase in apoptotic and necrotic cells compared to the untreated controls (SCC-4 72.4% vs 6.8%; SCC-25 76.9 vs 1.2%; all p<0.05). Quadrants 1(Q1) + Quadrants 2(Q2) was indicated as the late apoptotic and necrotic cells, Quadrants 3(Q3) as the living cells, and Quadrants 4(Q4) as the apoptotic cells (Fig. 3A). Moreover, both cells treated with 6-shogaol (10 mM) for 1, 2, 4, and 8 hours also significantly increased the level of cleaved caspase 3, a marker of the apoptosis development, compared to the untreated controls (Fig. 3B).

### ERK1/2 and p38 signaling regulate the 6-shogaol effect on SCC4 and SCC25 cells

**Figure 4:**
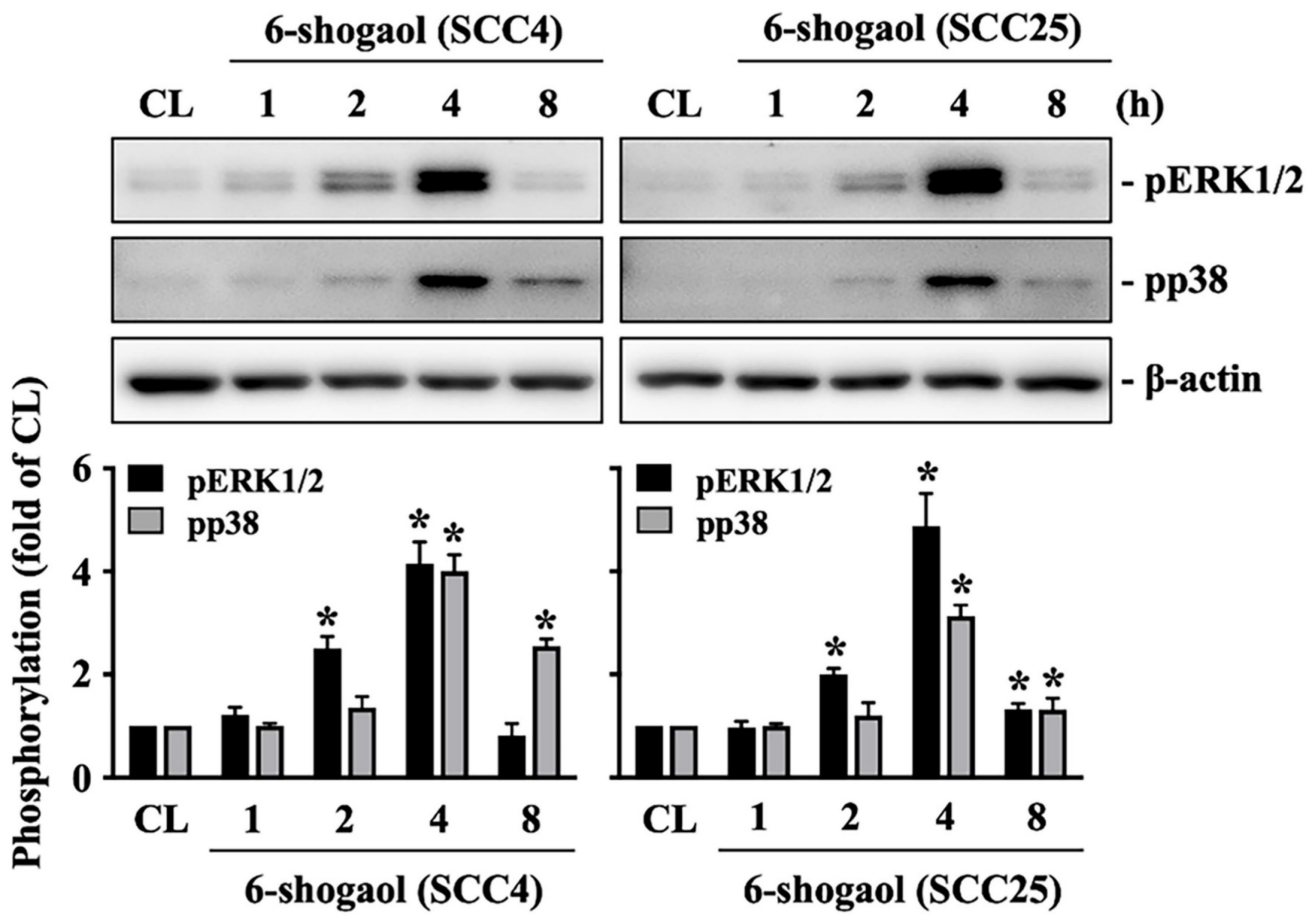
** 6-shogaol induces phosphorylations of ERK1/2 and p38 signaling in SCC4 and SCC25 cells.** SCC4 and SCC25 cells were kept as control or treated with 6-shogaol (10 μM) for 1, 2, 4, and 8 h and then the phosphorylations of ERK1/2 and p38 kinases were examined by Western blot. Results were representative of three independent experiments with similar results. Data are mean ± SEM from three independent experiments. *P < 0.05 versus untreated control.

**Figure 5:**
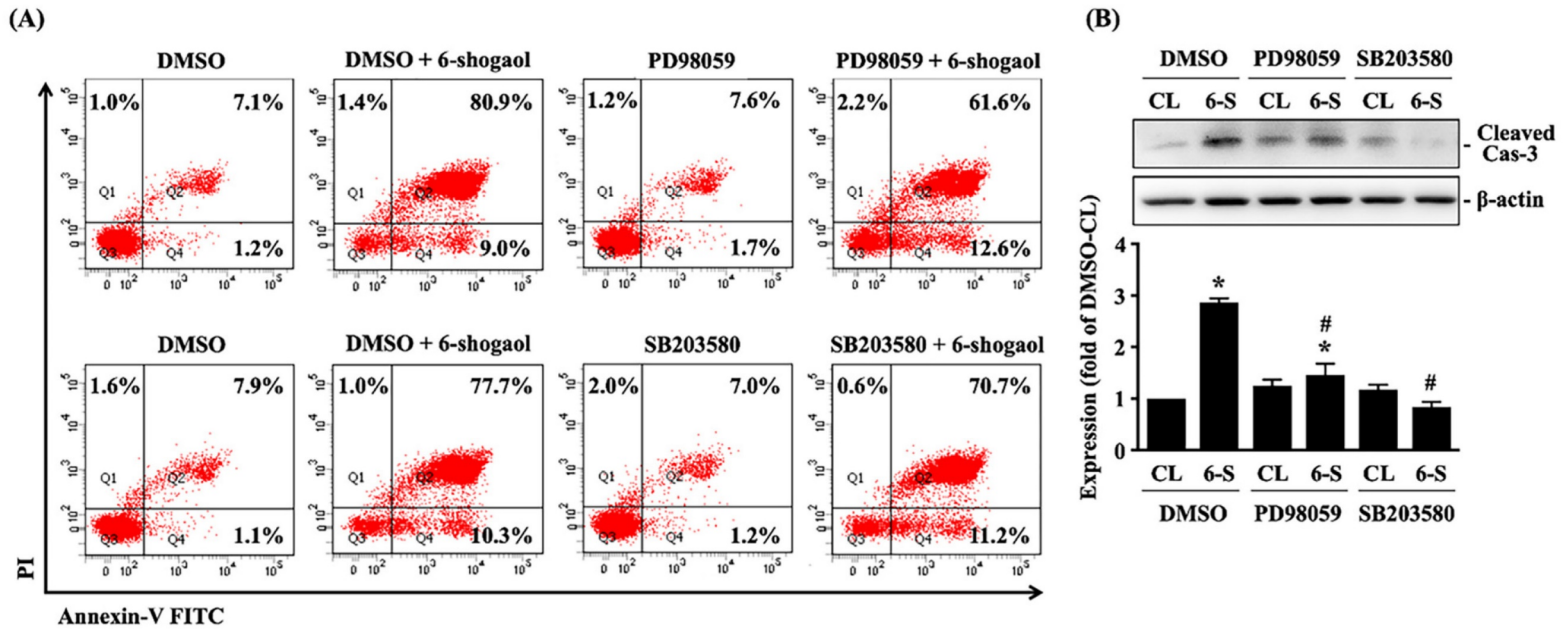
** 6-shogaol-induced apoptosis of SCC4 cells is mediated by ERK1/2 and p38 signaling. (A-B)** SCC4 cells were pretreated with vehicle (DMSO) or inhibitors of ERK1/2 (PD98059, 25 μM) and p38 (SB203580, 10 μM) for 1 h and then kept as control or treated with 6-shogaol (6-S, 10 μM) for 4 h (cleaved caspase 3 (Cas-3) level) and 6-shogaol (6-S, 30 μM) for 24 h (apoptosis). The apoptosis (A) and cleaved caspase 3 level (B) of treated cells were examined by PI/ Annexin V-FITC double stain and Western blot, respectively. (A) (Q1+Q2) was indicated as the late apoptotic and necrotic cells, Q3 was indicated as the living cells, and Q4 was indicated as the apoptotic cells. Data in (A, B) were shown as mean ± SEM from three independent experiments. Results in (B) were representative of three independent experiments with similar results. *P < 0.05 versus untreated control. #P < 0.05 vs. DMSO/6-shogaol-treated cells.

Both SCC4 and SCC25 cells were kept as control or treated with 6-shogaol (10 mM) for 1, 2, 4, and 8 hours and then the phosphorylations of ERK1/2 and p38 kinases were examined by Western blot. It showed that 6-shogaol induces rapid phosphorylations of ERK1/2 and p38 kinases in SCC4 and SCC25 cells within 2 hours and in a time-dependent manner compared to the untreated controls (Fig. 4). Moreover, SCC4 cells were further pretreated with vehicle (DMSO) or inhibitors of ERK1/2 (PD98059, 25 μM) and p38 (SB203580, 10 μM) for 1 hour and then kept as control or treated with 6-shogaol (10 mM) for 4 hours (cleaved caspase 3 level) and 6-shogaol (15 mM) 24 hours (cell cycle progression) and 6-shogaol (30 mM) for 24hours (apoptosis). The cell cycle progression, apoptosis, and cleaved caspase 3 level of treated cells were examined by PI stain, PI/ Annexin V-FITC double stain, and Western blot, respectively. It was shown that cells treated with 6-shogaol significantly induced G2/M phase arrest of the cell cycle in SCC4 cells, which resulted in a decrease in the G0/G1 phase and an increase in the G2/M phase (Table 3). Cells co-treated with ERK1/2 or p38 signaling inhibitors and 6-shogaol also decreased the distribution of the G0/G1 phase but increased the distribution of both S and G2/M phases in SCC4 cells (Table 3). Moreover, cells pretreated with ERK1/2 or p38 signaling inhibitors also block the 6-shogaol effect on inducing apoptosis (Fig. 5A) and reduce cleaved caspase 3 level (Fig. 5B) in SCC4 cells.

### 6-shogaol enhances the cytotoxic effect of cisplatin on SCC4 cells

**Figure 6:**
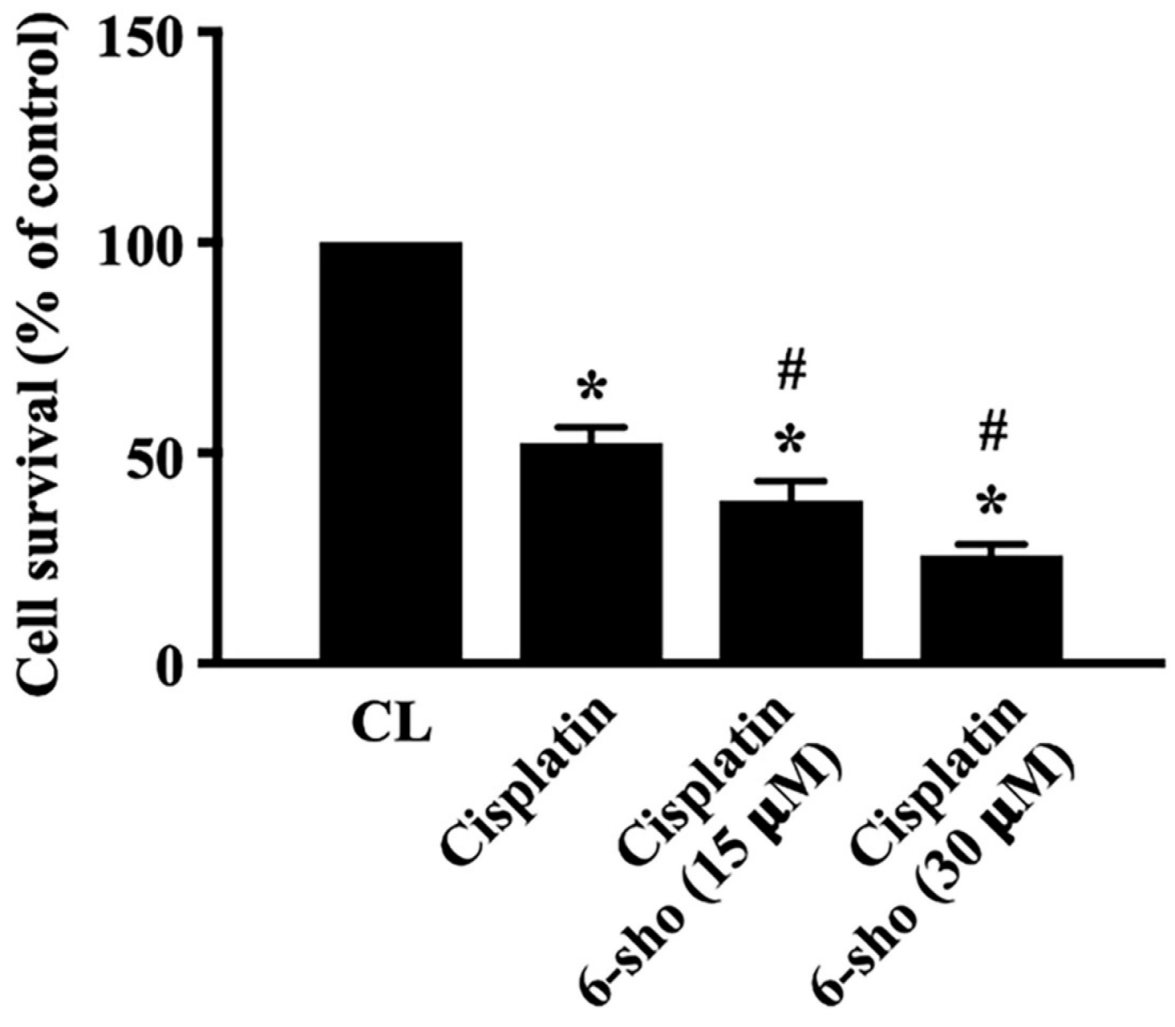
** 6-shogaol enhances the cytotoxic effect of cisplatin on SCC4 cells.** SCC4 cells were pretreated with DMSO or cisplatin for 1 h and then kept as control or treated with 6-shogaol (15 and 30 μM) for 24 h. The viability of treated cells was examined by MTT assay. Data were shown as mean ± SEM from three independent experiments. *P < 0.05 versus untreated control. #P < 0.05 vs. Cisplatin-treated cells.

Cisplatin is a useful drug for patients with HNSCCs. We finally investigated if combined treatment of cisplatin with 6-shogaol could enhance the cell death level of HNSCC cells. SCC4 cells were pretreated with DMSO or cisplatin for 1 hour and then kept as control or treated with 6-shogaol (15 and 30 mM) for 24 hours. The viability of treated cells was analyzed by MTT assay. It was shown that cisplatin induces ~50% cell death of SCC4 cells compared to the untreated controls and 6-shogaol could further enhance cisplatin cytotoxicity in SCC4 cells in a dose-dependent manner (Fig. 6).

## Discussion

**Figure 7:**
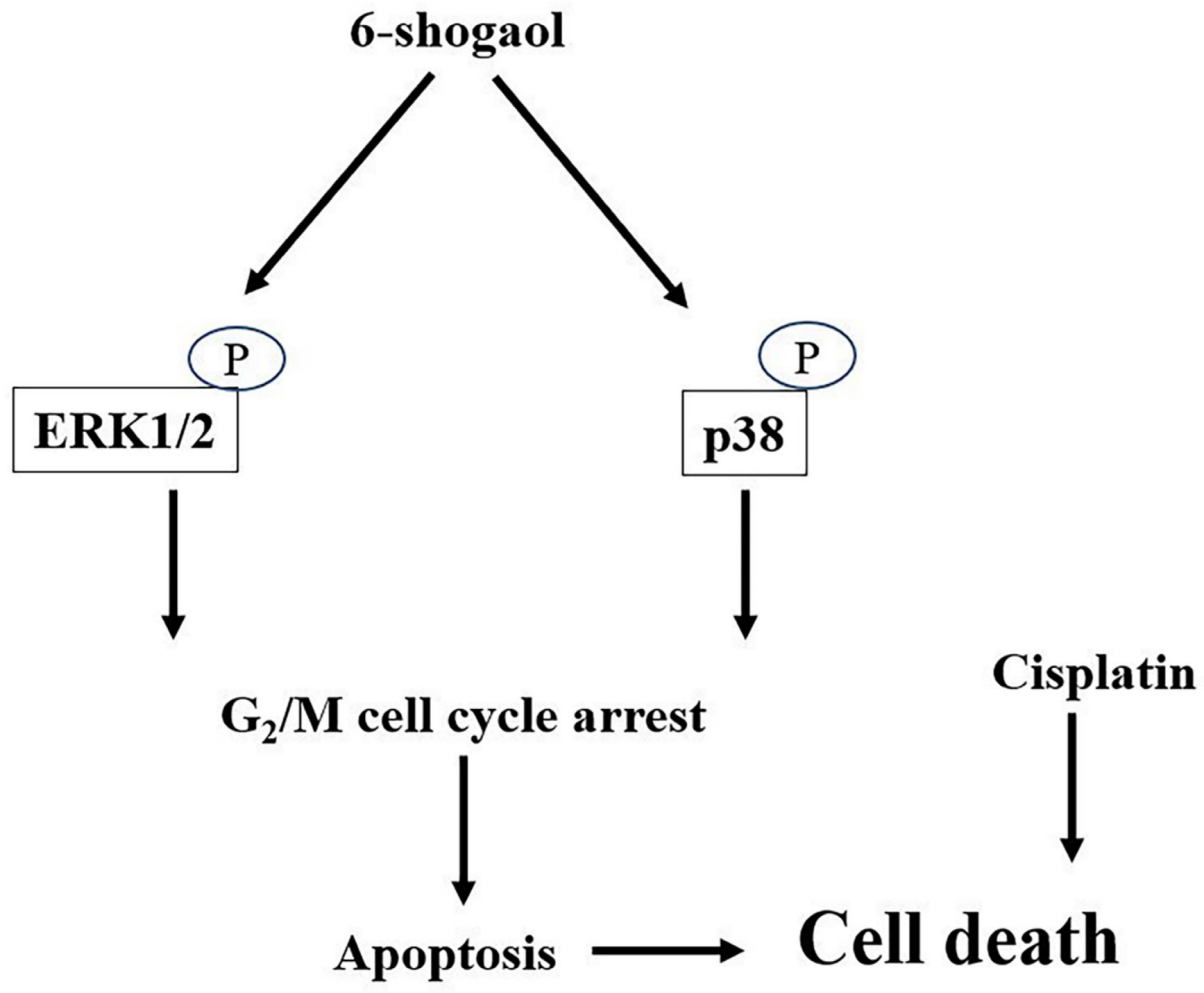
Schematic representation of the mechanisms of 6-shogaol in inducing cell death in HNSCC cells.

6-shogaol, one of the major ginger bioactive compounds, has been extensively investigated because of its effective bioactivity in anti-inflammation, anti-oxidation, anti-microbial, and even cancer-killing capability for many types of cancers. The present study was conducted based on these previous studies; the efficacy of 6-shogaol in retarding the survival of HNSCC cells, the possibility with reliable application in combination therapy of cisplatin with 6-shogaol were further explored in the future. Accordingly, the systematic experiments in the present study demonstrated that (i) 6-shogaol arrests cell cycle progression at the G2/M phase and initiates apoptosis in both types of HNSCC cells, i.e., SCC4 and SCC25, to result in their cell death; (ii) these responses are regulated by the ERK1/2 and p38 signalings; and (iii) combined treatment of cisplatin with 6-shogaol could enhance the cytotoxic sensitivity of HNSCC cells to cisplatin (summarized in Fig. 7).

Initially, we selected 10 μM as our LC 50 value and all western analysis were completed with 10 μM. But the difference between flow cytometry and cell cycle with 6-shogaol was not significantly changed with 10 μM. Therefore, we used 15 μM for the cell cycle and 30 μM for the flow cytometry study to get a more significant result.

In view of recent pharmaceutic development, vast works have been made efforts to screen and analyze the natural products from plants or many living organisms to find novel anticancer character. This is because most of the current drugs are chemically synthesized compounds and they usually elicit severe side effects, even though cancer could be eradicated effectively 21. Ginger is a traditional herb and is promoted to use in dietetic therapy for many years. The anticancer capability of ginger and its derivatives has been extensively investigated. These studies have elaborated the efficacy in (i) directly killing cancer cells; (ii) combining therapy with clinical drugs to reduce the dosage of these drugs and subsequent side-effect elicitation; (iii) lowering the occurrence of drug resistance 22-27. Recently, a new report proposed that 6-shogaol antagonizes the survival of HNSCC cells 20. Our present study confirmed this response and further elucidated the underlying mechanism and proposed a possibility of combined therapy of cisplatin with 6-shogaol in future HNSCC patients' treatment.

Cisplatin is a powerful clinical chemotherapeutic drug, which could be effectively applied to many cancer types, including lung, bladder, ovarian, and HNSCCs. However, the side effects of cisplatin had been considered as sticky trouble for patients 28,29. Therefore, accumulating study has tried to find a new adjuvant therapeutic strategy to reduce the cytotoxicity of cisplatin in normal cells or find a new combined therapeutic strategy to lower the cisplatin-using dosage. It has been suggested that ginger and its derivatives could be great potential candidates for this purpose. From recent animal studies, injection of cisplatin and oral administration of ginger has been found to significantly alleviate cisplatin-elicited cardiotoxicity, oxidative stress, and inflammation 28,30. Our present results support these previous ideas; however, the limitation of our study includes that there is no further cisplatin dosage experiment and no animal study to demonstrate our *in vitro* findings. And these would be our next elucidation in the future.

Cell cycle arrest at G0/G1 or G2/M phase has been considered a hint for the changes in cell status 31. When suffering from the damage, cells themselves could arrest their cell cycle progression to wait for the subsequent effective repair or death pathway. Facing the oppression of many clinical drugs, it was found that cancer cells might also arrest their cell cycle progression at the G2/M phase to seek the possibility of survival 32,33. Our results revealed that 6-shogaol contributes to the cell cycle arrest at the G2/M phase in both HNSCC cells before their cell death.

MAPKs are a family of enzymes that are currently known to regulate gene expression, mitosis, proliferation, motility, metabolism, and apoptosis 34. And they are particularly involved in the survival and proliferation of various cancer cells, making them a potential target for cancer therapy. Moreover, we also confirmed that ERK1/2 and p38 signaling played a important role in modulating the 6-shogaol-arrested cell cycle. Our data showed that blocking ERK1/2 and/or p38 signaling could decrease 6-shogaol-increased G2/M phase distribution of cell cycle but increase the DNA synthesis (S) phase distribution of cell cycle, which means that the proliferation of HNSCC cells indeed recovered ERK1/2 participates in G1/S and G2/M transitions. During G1/S, ERK1/2 regulates the transcription of cyclin D1 through the Fos protein family and Myc, impaired G2/M cell cycle arrest is associated with increased apoptosis 35. There is some evidence that the pro- and anti-apoptotic effects can be regulated by p38 signaling pathway 36.

However, ERK1/2 and p38 signaling pathways seem to regulate the apoptosis effect only partially on 6-shogaol-treated HNSCC cells. Moreover, ERK1/2 and p38 signaling block was shown to increase the endogenous cleaved caspase 3 level in HNSCC cells. According to these findings, we proposed that ERK1/2 and p38 signaling contributes to complex regulatory mechanisms in HNSCC, and these responses and further detailed mechanisms should be elucidated before the clinical application of 6-shogaol in HNSCC therapy. Until now, ginger is still a safe product. Adverse effects usually included heartburn, abdominal pain, nausea, diarrhea, bloating, epigastric distress, and gas 37. The recommended dosage range for ginger is 200mg to 2.5g of dry extract per day, which contains 1% to 4% shogaol 38.

However, there were some limitations in this study. There is only *in vitro* study, though the apoptosis regulated by ERK1/2 and p38 signaling pathway was seen, whether the anti-cancer effect of 6-shogaol, is still unknown in clinical practice. Further animal and prospective studies are needed to confirm the results of this study and clarify the mechanism of action.

6-shogaol is a promisingly potent phytochemical derivate of different edible plant sources against HNSCC cells. 6-Shogaol induces G2/M cell cycle arrest and reinforces apoptosis in SCC-4 and SCC-25 cells by MAPK signaling pathways. 6-shogaol, a natural medicine, is highly expected to be a useful, effective, and reliable choice for the adjuvant treatment of HNSCC.

## Conclusions

6-shogaol is a promisingly potent phytochemical derivate of different edible plant sources against HNSCC cells. 6-Shogaol induces G2/M cell cycle arrest and forces apoptosis in SCC-4 and SCC-25 cells by MAPK signaling pathways. 6-shogaol, a natural medicine, is expected to be useful for the treatment of HNSCC.

## Figures and Tables

**Table 1:** 6-shogaol results in a G2/M phase arrest of the cell cycle in SCC4 and SCC25 cells in a time-dependent pattern

Cell type	Duration (h)	Control, % of cells (mean ± SEM)			6-shogaol, % of cells (mean ± SEM)		
		G_0_/G_1_	Synthetic	G_2_/M	G_0_/G_1_	Synthetic	G_2_/M
SCC4							
	0	81.3 ± 2.3%	6.0 ± 0.8%	12.7 ± 1.1%			
	8	80.3 ± 1.5%	6.9 ± 1.1%	12.8 ± 1.4%	83.9 ± 1.2%	4.4 ± 1.4%	11.7 ± 1.2%
	24	74.0 ± 0.9%*	13.7 ± 1.2%*	12.3 ± 0.5%	71.2 ± 1.3%*	835 ± 2.3%^#^	20.3 ± 2.3%*^,#^
SCC25							
	0	82.1 ± 3.2%	5.4 ± 1.2%	12.5 ± 1.2%			
	8	84.3 ± 2.5%	5.8 ± 1.3%	9.9 ± 1.7%	81.1 ± 1.4%	5.0 ± 0.2%	13.9 ± 1.1%
	24	72.2 ± 2.4%	14.3 ± 1.1%*	13.5 ± 1.4%	69.6 ± 1.3%*	8.6 ± 2.5%^#^	21.8 ± 1.2%*^,#^

SCC4 and SCC24 cells were kept as control or treated with 6-shogaol (15 mM) for 8 and 24 h. The cell cycle progression of treated cells was examined by PI stain and flow cytometry analysis to show percentages of cells in the G0/G1, Synthetic, and G2/M phases of the cell cycle. Data are mean ± SEM from three independent experiments. *, P < 0.05 vs. control-0 h cells. #, P < 0.05 vs. control cells at the corresponding times.

**Table 2:** 6-shogaol results in a G2/M phase arrest of the cell cycle in SCC4 and SCC25 cells in a dose-dependent pattern

Cell type	Treatment (24 h)	Control, % of cells (mean ± SEM)		
		G_0_/G_1_	Synthetic	G_2_/M
SCC4				
	Control	60.0 ± 1.3%	16.5 ± 1.7%	23.5 ± 0.3%
	6-shogaol 15 μM	44.2 ± 1.5%*	20.5 ± 1.4%	35.3 ± 2.2%*
				
	6-shogaol 30 μM	50.2 ± 2.1%*	18.9 ± 1.2%	31.0 ± 2.9%*
SCC25				
	Control	55.7 ± 2.6%	22.0 ± 3.2%	22.2 ± 2.9%
	6-shogaol 15 μM	45.0 ± 1.3%*	22.3 ± 0.4%	32.7 ± 1.7%*
	6-shogaol 30 μM	49.7 ± 0.9%*	17.5 ± 1.2%	32.8 ± 1.3%*

SCC4 and SCC25 cells were kept as control or treated with 6-shogaol (15 and 30 mM) for 24 h. The cell cycle progression of treated cells was examined by PI stain and flow cytometry analysis to show percentages of cells in the G0/G1, Synthetic, and G2/M phases of the cell cycle. Data are mean ± SEM from three independent experiments. *, P < 0.05 vs. control cells.

**Table 3:** 6-shogaol-induced G2/M arrest in SCC4 cells is mediated by ERK1/2 and p38 signaling

SCC4	Control, % of cells (mean ± SEM)			6-shogaol, % of cells (mean ± SEM)		
	G_0_/G_1_	Synthetic	G_2_/M	G_0_/G_1_	Synthetic	G_2_/M
DMSO	62.3 ± 1.2%	18.2 ± 1.1%	19.5 ± 0.7%	42.3 ± 2.1%*	19.8 ± 0.7%	37.9 ± 1.7%*
PD98059	63.5 ± 2.0%	16.9 ± 1.6%	20.5 ± 0.9%	34.6 ± 3.1%*^,#^	27.8 ± 2.2%*^,#^	37.6 ± 2.2%*
DMSO	59.0 ± 0.6%	17.0 ± 1.2%	24.0 ± 0.7%	23.7 ± 0.8%*	19.4 ± 1.4%	56.9 ± 2.3%*
SB203580	58.8 ± 0.9%	17.8 ± 1.1%	23.4 ± 1.7%	31.2 ± 1.3%*^,#^	26.0 ± 1.1%*^,#^	42.8 ± 2.4%*^,#^

SCC4 cells were pretreated with vehicle (DMSO) or inhibitors of ERK1/2 (PD98059, 25 μM) and p38 (SB203580, 10 μM) for 1 h and then were kept as control or treated with 6-shogaol (15 mM) for 24 h. The cell cycle progression of treated cells was examined by PI stain and flow cytometry analysis to show percentages of cells in the G0/G1, Synthetic, and G2/M phases of the cell cycle. Data are mean ± SEM from three independent experiments. *P < 0.05 vs. corresponding control cells. #P < 0.05 vs. DMSO/6-shogaol-treated cells.
